# Editorial: Nutrition and sustainable development goal 3: good health and wellbeing

**DOI:** 10.3389/fnut.2024.1542307

**Published:** 2025-01-09

**Authors:** Elezebeth Mathews, Elena Ibáñez, Alejandro Cifuentes, Miroslava Atanassova

**Affiliations:** ^1^Department of Public Health and Community Medicine, Central University of Kerala, Kasaragod, India; ^2^Laboratory of Foodomics, Institute of Food Science Research, Madrid, Spain; ^3^Møreforsking, Volda, Norway

**Keywords:** malnutrition, nutritional epidemiology, nutrition transition, SDG 3, chronic disease

Nutrition is recognized to play a pivotal role in human life and its existence. Investing in nutrition yields significant returns for individuals—enhancing school performance and workforce productivity—and societies—enhancing national economic growth. This creates a ripple effect that extends beyond individual health. Nutrition is deeply interconnected with at least 12 of 17 Sustainable Development Goals (SDGs) and is crucial for achieving these goals.

In this Research Topic on Nutrition and Sustainable Development Goal 3: Good Health and Wellbeing, we provide recent evidence on the role of nutrition in promoting good health and wellbeing, as well as the future of food. SDG 3 addresses a wide range of disease conditions across all age groups, encompassing maternal and child health, non-communicable diseases, communicable diseases such as AIDS, tuberculosis, malaria, neglected tropical diseases, hepatitis, water-borne diseases, and substance use disorders. The Global Burden of Diseases study in 2017 attributed 11 million (95% uncertainty interval [UI] 10–12) deaths and 255 million (234–274) DALYs to dietary risk factors (1).

With the economic, demographic, and epidemiological changes in both low- and middle-income countries, there has been a drastic shift in dietary consumption and energy expenditure, commonly referred to as the “nutrition transition” (2). This transition has contributed to the burden of malnutrition, characterized by overnutrition, undernutrition, and micronutrient deficiencies often called “hidden high hunger.” Sub-optimal intake of healthy food is one of the major drivers of non-communicable diseases (NCDs), such as diabetes, obesity, and other metabolic disorders, as well as cardiovascular diseases. Undernutrition, mostly due to poverty, hunger, and nutrition illiteracy, results in stunted growth, poor maternal and child health outcomes, and greater susceptibility to communicable diseases. Globally, malnutrition remains a persistent challenge. Several regions across the world are witnessing the paradoxical coexistence of undernutrition and overnutrition. Kumma et al. in a study involving 2,483 Ethiopian participants aged 25–64 years, reported changing dietary patterns within the Ethiopian population, with the co-existence of Western, traditional, and healthy dietary patterns. The study highlighted the association between these changing dietary patterns and one or more cardiovascular risk factors, with Western diet consumers being at a higher risk compared to traditional diet consumers. The authors emphasized the need to promote healthy and traditional dietary patterns, along with physical activity, to address the rising incidence of cardiovascular diseases in Ethiopia. Similarly, Chen et al. identified distinct dietary patterns, namely vegetable-rich, animal-food, and prudent dietary patterns, using data from the Nutrition and Health in Southwest China (NHSC) 2013–2018 survey. The prudent dietary pattern, characterized by ethnic foods, whole grain products, fruits, eggs, and dairy and wheat products, was associated with lower systolic and diastolic blood pressure among the Southwest Chinese population. Adeba et al. in their study in Western Ethiopia, reported high prevalence of unhealthy dietary practices (73.3%), which were found to be associated with low income, being unmarried, daily meal frequency, and poor knowledge about a healthy diet.

Sustainable Development Goals envisage holistic development and well-being from the perspective of a life-course approach. Good Nutrition lays the foundation of health, that begins at the preconception stage to proper growth and development in childhood and adolescence, and good health and wellbeing in the adulthood. Adequate nutrition during the first 1,000 days of life (3) is critical for physical growth and cognitive development. Childhood undernutrition causes nearly 45% of deaths among children under the age of 5 (3) (WHO) and results in long-term irreversible effects, including impaired physical growth, recurrent infections, and cognitive underdevelopment (4). In a randomized intervention study by Gsoellpointner et al. in Vienna, nutrient supplementation through the early introduction of solid foods improved zinc, calcium, and phosphorus intake in very low birth weight (VLBW) infants during the first year of life. The authors further recommended prolonged iron and vitamin D supplementation for least 12 months to meet the recommended levels. Larson et al., in a meta-analysis of trials on egg consumption and growth in children, found significant improvements in the height and weight of children who consumed eggs compared to the control group, suggesting that eggs are an affordable nutritional option in low- and middle-income countries. Beyond dietary supplementation, significant attention must be given to addressing the social determinants of health to combat malnutrition. A study by Sanin et al. explored the factors influencing childhood undernutrition in vulnerable regions of Bangladesh using data from the Bangladesh Demographic and Health Survey (2007–2018). A decadal reduction in stunted growth and underweight among children under five was observed. The study found that urban residence, the child's age and gender, morbidity, maternal body mass index, maternal and paternal education, decision-making ability, use of contraceptives, the occurrence of domestic violence, antenatal care, mode of delivery, birth interval, and geographic region were all associated with childhood malnutrition.

Adolescence is a transitional phase of human life, marking the shift from childhood to adulthood, and is characterized by physical, mental, and psychosocial changes. Survivors of childhood malnutrition often experience chronic energy deficiency (CED) and more than one type of malnutrition. Yulia et al. in a study conducted in Indonesia, found that half of the adolescents in urban (54%) and rural (61.7%) areas were at risk for CED and consumed inadequate macronutrients. The double burden of malnutrition (DBM), characterized by both overnutrition and undernutrition, is predominant in low- and middle-income countries due to the complex interaction of poor nutrition, biological factors, and environmental and social influences across the life course. A school-based study by Getacher et al. among individuals aged 10–19 years in Ethiopia found a DBM prevalence of 21.5% (14.8% thinness and 6.7% overweight/obesity), which was associated with age, gender, type of school, dietary diversity, meal frequency, home gardening practice, illness history, and knowledge of nutrition. Multi-sectorial interventions addressing the two contradictory nutrition paradigms are crucial for alleviating the growing burden of malnutrition. Healthy eating among adolescents can be promoted by creating supportive environments for healthy eating and physical activity in educational settings, starting from the early years. Preschool and school educators need to be trained with the necessary skills and expertise to support the holistic development of children, including fostering healthy behaviors related to diet and physical activity. Educational institutions are platforms for molding healthy future generations. In a study by Lafave et al., the CHEERS eHealth program improved nutrition and physical activity practices within early childhood education and care (ECEC) centers. They found that educators' personal nutrition-related knowledge, attitude, and behaviors were positively associated with their self-assessments of the nutrition environment and practices in ECEC centers. The CHEERS survey's Food Served subscale further showed a positive correlation with the objective measures of the EPAO-Foods Provided and Nutrition Policy subdomains.

Maintaining an optimal body mass index is critical at all stages of life. Globally, 49% of adults are overweight or obese. Several studies continue to explore the diet-related factors that contribute to obesity, as well as effective nutritional therapies to prevent it. Cattaneo et al., in an RCT, observed that a 4-week restricted Mediterranean diet was effective in improving anthropometric and blood parameters among participants with severe obesity. The authors recommend targeting taste as a new approach to prevent the risk of therapeutic failure. Another randomized controlled trial by Hooshiar et al., among women with obesity and overweight in Iran, showed that the alternate day modified fasting (ADMF) diet was effective in weight reduction [−5.23 (1.73) vs. −3.15 (0.88); *p* < 0.001] and body mass index [−2.05 (0.66) vs. −1.17 (0.34); *p* < 0.001] compared to the daily calorie restriction (CR) diet. There were significant improvements in sleep and daytime dysfunction. Future studies need to explore the long-term effects of the ADMF diet on overweight/obesity. Conversely, low BMI was found to be inversely related to mortality (5). A study by Ishikawa et al., found improvements in the physical and mental health scores of health-related quality of life within 12 weeks of medium-chain triglycerides (MCT) supplementation and moderate-intensity walking exercise among sedentary older adults aged 60–74 with low BMI values (< 24 kg/m^2^).

Nutritional epidemiological studies have established irrefutable evidence on the relationship between diet and chronic diseases, and several observational studies, trials, and meta-analyses have examined the complex relationship between diet and diseases. A prospective cohort study by Kityo et al. in Korea among 13,568 adults included in the Health Examinees-Gem (HEXA-G) reported high intake of processed red meat to be a risk factor for all-cause mortality [men: hazard ratio (HR) 1.21, 95% CI 1.07–1.37; women: HR 1.32, 95% CI 1.12–1.56]. An increased risk of all-cause mortality (HR 1.21, 95% CI 1.05–1.39) and cancer mortality (HR 1.24, 95% CI 1.03–1.50) was observed in women with high intake of organ meat. Moderate intake of pork belly was associated with a reduced risk of all-cause mortality in men (HR 0.76, 95% CI 0.62–0.93) and women (HR 0.83, 95% 0.69–0.98), but high intake was associated with an increased risk of CVD mortality in women (HR 1.84, 95% CI 1.20–2.82). Xing et al., in a mendelian randomization analysis on a European cohort, showed genetically determined tea intake to have a causal impact on total body bone mineral density (TB-BMD), with an odds ratio (OR) of 1.204 (95% CI: 1.062–1.366, *p* = 0.004), especially in the age group of 45–60 years (OR = 1.360, 95% CI: 1.088–1.700, *p* = 0.007). Tea consumption was found to increase bone density and reduce the risk of osteoporosis in the age group of 45–60 years within the European population. The findings of a large-scale prospective cohort study in the US by Qi et al., involving 101,190 participants with a median follow-up of 12.2 years, suggested that dietary glycaemic index was associated with a higher risk for renal cancer (HR Q3 vs. Q1: 1.38; 95% CI: 1.09–1.74). Further studies are recommended in other populations to establish dietary GI as a modifiable risk factor for renal cancer prevention. Torabynasab et al. in a systematic review and meta-analysis of cross-sectional studies, found a protective effect of dietary caffeine against the development of depression, with no evidence linking tea consumption. The authors recommended further longitudinal studies to establish the causal relationship between coffee, tea, and caffeine and the risk of depression. Al-Maweri et al., in an updated meta-analysis, found a significant association between low serum levels of vitamin D and the risk of recurrent aphthous stomatitis (mean difference = – 8.73, 95% CI: – 12.02 to – 5.44) and recommended screening and supplementation of Vitamin D for the prevention and treatment of aphthous stomatitis. Wang X. et al. observed a relationship between vitamin K and metabolic dysfunction-associated fatty liver disease (MAFLD) among individuals from the United States using the National Health and Nutrition Examination Survey 2017–2018. The -MAFLD population had lower vitamin K intake than the non-MAFLD population, suggesting a protective effect. Further prospective studies or intervention trials are warranted to establish the causal relationship.

Recent advances in molecular epidemiology have paved the way for a greater understanding of the molecular determinants of nutritional imbalances and disorders. The Oxidative Balance Score (OBS), which assesses the impact of diet and lifestyle on oxidative stress, has been linked to lower risks of metabolic syndrome. Liu and Chen found the OBS to be inversely related to the risk of NAFLD among 6,341 adult participants using the US National Health and Nutrition Examination Survey 1999–2018. On a similar note, Park et al. found an inverse relationship between the OBS and metabolic syndrome among 2,735 adults over 19 years and 5,807 adults aged 40–69 years using data from the Korean National Health and Nutritional Examination Survey (KNHANES) and the Korean Genome and Epidemiology Study (KoGES), respectively. These findings suggest the benefits of a healthy lifestyle for the prevention of metabolic syndrome. Wang P. et al. observed a 24% decrease in the risk of non-cancer mortality (aHR = 0.76, 0.60–0.92) among 5,009 cancer patients who had higher dietary total antioxidant capacity (DAC) but no significant effect on all-cause or cancer mortality. Higher dinner DAC, rather than breakfast or lunch DAC, was associated with a 21% lower risk of all-cause mortality (aHR = 0.79, 95% CI: 0.65–0.98) and a 28% lower risk of non-cancer mortality (aHR = 0.72, 95% CI: 0.57–0.90). The authors emphasized the importance of advocating for DAC consumption at dinner to reduce mortality risk in cancer survivors.

Nutraceuticals—nutrient-based supplements and plant-derived products—are gaining recognition for their health benefits. Derbo et al. in a community-based study in Ethiopia, found that 49.6% of participants consumed *Moringa stenopetala* leaves (locally known for reducing the risk of malnutrition, low birth weight, and anemia) during pregnancy. Factors associated with the consumption included younger age (below 24 years), rural residence, antenatal care attendance, a history of contraceptive use, and having good knowledge about the importance of *Moringa stenopetala*. Studies have shown that omega-3 fatty acids (Gui et al.) improve nutritional status and reduce chronic inflammation in cancer patients, while flaxseed supplementation (Musazadeh et al.) had no significant effect on sex hormone levels. Li et al., in a meta-analysis of RCTs, found that oral intake of fruits or fruit extracts led to significant improvements in skin hydration and a decrease in transepidermal water loss (TEWL). Wang N. et al. in animal studies, reported that consuming fermented wax gourd, a traditional food of Eastern China, enhanced the presence of beneficial probiotics and reduced pathogenic *Helicobacter sp*. in the mouse gut.

Synbiotics, which combine probiotics and prebiotics, are another promising area of research. However, a study by Talebi et al. among women with polycystic ovary syndrome (PCOS) yielded mixed results, highlighting the need for more rigorous trials.

Nutritional literacy is recognized to have a positive influence on behavior change and the adoption of healthy eating practices. However, many populations, especially those living with HIV or in low-income regions, still face significant barriers to proper nutrition. Gemede et al. observed that poor nutritional knowledge and practices among HIV-positive adults in Ethiopia were 74.9 and 69.1%, respectively. Nutritional knowledge was associated with factors such as educational level, monthly income, occupation, and marital status. A study by Zhang et al. showed that knowledge, attitude, and practices regarding oil and salt intake were relatively poor, with factors such as region, ethnicity, urban and rural residence, education, taste preference, and the prevalence of chronic diseases influencing the oil- and salt-related KAP scores. Yang et al., in a study among elderly populations in rural China, found that higher dietary indices were associated with better quality of life scores, highlighting the important role of a healthy diet in improving overall wellbeing.

The COVID-19 pandemic has had both short- and long-term effects on health, particularly affecting eating habits and lifestyle behaviors. Rafraf et al., in their study, found that university students experienced weight gain, reduced physical activity, and worsened sleep quality during the pandemic. In Indonesia (Fatmah), food insecurity worsened, affecting many families' ability to access nutritious foods. These findings highlight the need to address nutritional needs during and after a pandemic.

At the population level, nutrition policies play a crucial role in promoting health. Kirk et al. examined the impact of the Affordable Care Act (ACA) on nutrient consumption in the United States, using data from the National Health and Nutrition Examination Survey (NHANES). The study found that the intake of micronutrients from nutrient-dense foods, such as fruits and vegetables, did not change significantly after the ACA was implemented. However, there was an increase in the use of nutritional supplements after the ACA (*p* = 0.05), particularly for magnesium (OR = 1.02), potassium (OR = 0.76), vitamin D (both D2 and D3, OR = 1.34), vitamin K (OR = 1.15), and zinc (OR = 0.83). This trend was observed in both the general population and specific subgroups, including cancer survivors and Medicaid recipients. Given the connection between increased supplement use and expanded insurance coverage, the authors call for further research to better understand how broader access to nutritional supplements might influence the intake of both micronutrients and macronutrients, helping to meet daily recommended nutritional requirements.

Achieving SDG 3 requires a comprehensive approach to nutrition that extends beyond health to include sustainable food systems and equitable food distribution. Advances in food production and technology are essential for improving food quality and ensuring access to healthy food. Unsustainable agricultural practices, food waste, and inequitable food distribution exacerbate environmental degradation and hinder equitable access to nutritious foods. To address this disparity, it is critical to focus on a new model of sustainable food production to improve food systems. Wang and Zhang in a review, shed light on the “big food view” to enhance food production, improve quality and diversity through scientific and technological innovation, and enable access to healthy food for better living.

The path to achieving SDG 3 is closely linked to the global commitment to improving nutrition. Without addressing the root causes of malnutrition and fostering sustainable food systems, the goal of ensuring good health and wellbeing for all will remain unmet. As we progress toward 2030, nutrition must not be merely an agenda item but the core of efforts to achieve health equity and environmental sustainability. A well-nourished population is the foundation of a prosperous and thriving world.
